# Whooping cough hospitalisations among patients with chronic conditions: a retrospective analysis of incidence and costs, France, 2008 to 2020

**DOI:** 10.2807/1560-7917.ES.2026.31.32.2500561

**Published:** 2026-08-13

**Authors:** Juan C Vargas-Zambrano, Mickael Arnaud, Ana Antunes, Khalil Karzazi, Johanna Despres, Regis Verdier, Florence Boisnard, Jerome Jund, Cecile Janssen, Didier Pinquier, Denis Macina

**Affiliations:** 1 Global Medical, Vaccines, Sanofi, Lyon, France; 2 Real World Solutions, IQVIA, Bordeaux, France; 3 Real World Solutions, IQVIA, Lisbon, Portugal; 4 Real World Solutions, IQVIA, Paris, France; 5 France Medical, Vaccines, Sanofi, Lyon, France; 6 Service d’information et d’Evaluation médicale, Centre Hospitalier Annecy Genevois, Epagny Metz-Tessy, France; 7 Service d'Infectiologie, Centre Hospitalier Annecy Genevois, Epagny Metz-Tessy, France; 8 Service de Pédiatrie Néonatale et Réanimation, University of Rouen Normandy, CHU Rouen, Hôpital Charles Nicolle, Rouen, France

**Keywords:** Pertussis, Hospitalisation, Asthma, COPD, immunocompromising conditions, whooping cough, chronic conditions

## Abstract

**BACKGROUND:**

In European countries, including France, the incidence and costs of whooping cough in people with chronic conditions remains understudied.

**AIM:**

We aimed to estimate cumulative incidence and costs of whooping cough hospitalisations in children, adolescents and adults with chronic conditions in France.

**METHODS:**

We extracted data from patients hospitalised with whooping cough with or without underlying chronic conditions, asthma, chronic obstructive pulmonary disease (COPD), diabetes and immunocompromising conditions between 2008 and 2020 using the Système National des Données de Santé (SNDS).

**RESULTS:**

Among the 10,893 patients hospitalised with whooping cough in 2008–2020, asthma and/or COPD were the most common chronic conditions. Of the 7,212 children and adolescents hospitalised, 1,263 (17.5%) had asthma and 68 (0.9%) had an immunocompromising condition. Of the 3,681 adults, 2,297 (62.4%) had asthma and/or COPD, 594 (16.1%) had an immunocompromising condition and 454 (12.3%) had diabetes. In children and adolescents, the highest cumulative incidence ratio (CIR) was among patients with an immunocompromising condition (adjusted CIR = 3.6; 95% confidence interval (CI): 2.8–4.6) whereas in adults, patients with asthma and/or COPD had the highest adjusted CIR (18.6; 95% CI: 17.4–19.9). Median hospitalisation costs were the highest for patients with an immunocompromising condition (EUR 4,401). No significant changes in healthcare resource utilisation were observed before and after whooping cough hospitalisation in adults.

**CONCLUSION:**

Chronic conditions are associated with higher cumulative incidence of whooping cough-related hospitalisation in France. This highlights the importance of reducing whooping cough circulation, through strengthening vaccination programmes, particularly for those with chronic conditions.

**Key public health message**

**What did you want to address in this study and why?**

Whooping cough is a highly contagious bacterial respiratory disease that can be prevented through vaccination. We wanted to investigate the number of patients hospitalised for whooping cough and costs for hospitalisation for patients with chronic conditions such as asthma, chronic obstructive pulmonary disease (COPD), diabetes or weakened immune systems. We used French national healthcare data from 2008 to 2020.

**What have we learnt from this study?**

Of the almost 11,000 patients hospitalised with whooping cough, those with chronic conditions had higher rates of hospitalisation. Children and adolescents with a weakened immune system and adults with asthma or COPD had the highest hospitalisation rates. Hospitalisation costs were highest for those with weakened immune system (EUR 4,401).

**What are the implications of your findings for public health?**

Asthma, COPD and weakened immune system seem to increase hospitalisation risk when such individuals get whooping cough, regardless of age. Therefore, improving disease awareness and vaccination coverage is essential, especially for people with existing health conditions, to reduce disease spread and lower healthcare costs.

## Introduction

Pertussis or whooping cough is a highly transmissible respiratory infectious disease mostly caused by the bacterium *Bordetella pertussis* [1], while other species of the *Bordetella* genus (i.e. *B. holmesii* and *B. parapertussis*) can also cause similar disease, although less frequently and less severe [2].

Whooping cough can occur at any age; however, the disease is most common and most severe in infants (aged < 1 y) [1,3,4]. It is often under-recognised [5-7], under-diagnosed and under-reported in adolescents and adults, as these bacteria are not usually tested for in this population. Due to the higher severity of the disease, infants with whooping cough are more often hospitalised than adults [8]. However, in adults, 3% of patients with whooping cough are hospitalised, which increases to 12% in older adults [9].

Adults and adolescents with whooping cough usually present symptoms such as persistent cough [10]. In a previous epidemiological study, the highest case fatality rate (1.6%) was observed among adults aged ≥ 50 years hospitalised with pertussis, despite the greatest need for intensive care in infants younger than 3 months [11]. Importantly, 76% of fatal cases with whooping cough among adults aged ≥ 50 years had at least one underlying medical condition, highlighting the critical role of chronic conditions in the risk of death.

Adult patients with whooping cough often have pre-existing chronic conditions such as asthma, thereby suggesting that these factors may be crucial in increasing the severity of whooping cough in these individuals [12-14]. In a study conducted in the United States (US), at least one pre-existing chronic condition was recorded in 32.6% of patients hospitalised with whooping cough regardless of age, and more than 86% in adults aged ≥ 21 years [8]. In adults, underlying chronic conditions, particularly respiratory diseases, such as asthma and chronic obstructive pulmonary disease (COPD), have been associated with higher likelihood of whooping cough [15].

In France, whooping cough was a mandatorily notifiable disease until 1986. After the implementation of whooping cough vaccination in 1959, whooping cough cases declined. The resulting low annual incidence of whooping cough indicating a likely decline in the circulation of *B. pertussis*, as well as under-diagnosis and/or under-notification of cases, may have led to the suspension of mandatory surveillance [16]. Therefore, for surveillance of whooping cough, the country is dependent on sentinel surveillance networks of paediatric hospitals and healthcare, limiting the quantification of the number of cases across all ages nationwide [16]. The Système National des Données de Santé (SNDS; the French National Health Data System) covers the healthcare claims data of the French population from multiple databases [17,18]. The SNDS allows accessing information on the hospitalised cases of whooping cough.

We have previously published the incidence and costs of hospitalisations of whooping cough in France between 2008 and 2020: showing three outbreaks (2009, 2012–2013 and 2017–2018), higher cumulative incidence in young infants (aged < 5 months) and older adults (≥ 65 years) [19] and the effectiveness of pertussis vaccines against *B. pertussis* in children aged < 5 years [20]. Here we aimed to study the cumulative incidence, characteristics, healthcare resource utilisation (HCRU) and costs for patients hospitalised with whooping cough diagnoses (including all *Bordetella* species) with chronic conditions.

## Methods

### Study design

We conducted a retrospective open cohort study among French residents between January 2008 and December 2020 using data from SNDS and using hospitalisation with whooping cough as main outcome. These persons were followed until one of the following events occurred: death, exit from the cohort or end of study period (31 December 2020). Results were then stratified by the following pre-existing chronic conditions: asthma, COPD, diabetes and immunocompromising conditions, and contrasted to those without these conditions. As this study complements previously published analyses of the dataset, we used the same methodology, including data source and case definition as in [19] and briefly explained hereafter.

### Data sources

We received data from the following databases in SNDS: Données de Consommation Inter-Régime (DCIR) for healthcare resource utilisation, demographic and chronic conditions information, Programme de médicalisation des systèmes d’information (PMSI), a hospital discharge database with admission and discharge details, and Echantillon Généraliste de Bénéficiaires (EGB), which provides linked data from DCIR and PMSI for a 1/97th random sample representative of the population of France.

To comply with the principle of minimising the data to be processed, only data from DCIR and PMSI for all patients hospitalised with whooping cough between 2008 and 2020 were extracted. Additionally, the EGB database was used to estimate the size of the populations with chronic conditions in France, which served as denominators for calculating cumulative incidences. Due to data privacy requirements when ≤ 10 patients were identified for a condition or age group, we used ranges instead of exact numbers to avoid secondary derivation of the exact values.

### Whooping cough case definition

The definition of whooping cough used in this study is based on the International Classification of Diseases, Tenth Revision (ICD-10) diagnosis codes, as described in [19] including codes for all *Bordetella* species causing whooping cough: ICD-10 A37.0 (*B. pertussis*), A37.1 (*B. parapertussis*), A37.8 (other *Bordetella* species) and A37.9 (unspecified). Codes A37.0 and A37.9 were categorised as *B. pertussis* while A37.1 and A37.8 as other *Bordetella* species. Severe disease was defined as intensive care unit (ICU) admission, including any of the following: admission to resuscitation, intensive care, continuous monitoring units and short-stay emergency observation or death during hospitalisation. This is a broad definition, as not all patients admitted to short-stay emergency observation units require critical-care-level management.

### Chronic conditions

The chronic conditions included in this study were asthma, COPD, diabetes and immunocompromising conditions. We included asplenia, hereditary immune deficiency, HIV/AIDS, solid tumours, malignant haemopathies, solid organ transplantation, haematopoietic stem cell transplantation, autoimmune or non-respiratory chronic inflammatory diseases treated with immunosuppressants or corticosteroids and nephrotic syndrome in immunocompromising conditions.

The chronic conditions were captured through algorithms combining hospital and/or long-term disability diagnoses, treatments used, and procedures and laboratory tests performed. These conditions were identified through a previously created list of medicinal products based on anatomical therapeutic chemical (ATC) and ICD-10 diagnosis codes. The algorithms for capturing asthma and COPD relied on the use of specific treatments which can be indicated for both diseases. However, because SNDS does not provide information on the indication for which a treatment is used, there may be a misclassification of cases, as several medicines can be used for asthma and COPD. Hence, the cases where it was impossible to determine which of the two diseases the patient had were categorised as the asthma and/or COPD group. Due to the rarity of COPD in children and adolescents, COPD was not reported for children and adolescents. Therefore, results for children and adolescents under the asthma and/or COPD category include asthma only. In some analyses presenting aggregated data across all age groups, the full asthma and/or COPD algorithm output was retained, including children and adolescents, which may result in a higher number of cases compared with the sum of the individual age-group breakdowns.

### Statistical analysis

#### Cumulative incidence

Analyses here followed the same approach of the statistical analyses done in the previous publication [19], however, there were important differences to determining the cumulative incidence in each chronic condition. The cumulative incidences of hospitalisation due to whooping cough between 2008 and 2020 among individuals with/without each chronic condition of interest were estimated using the total number of hospitalised whooping cough cases with/without the chronic condition as a numerator and the sum of the annual number of French residents with/without this condition identified from EGB and then extrapolated to whole France using census data as the denominator. The statistical unit for incidence analyses was the patient. Precisely, only the first hospitalisation observed between 2008 and 2020 at the patient-level data was considered for these analyses. Cumulative incidences were stratified by age group, and severity and per 100,000 inhabitants.

To compare the cumulative incidences in cases with and without the chronic conditions, negative binomial regressions adjusting for the cyclical pattern of whooping cough were used to determine cumulative incidence ratios (CIRs).

#### Costs

We included all hospitalisations recorded between 2011 and 2020 in the analyses. Of note, transfer to another service or hospital was considered as part of the same hospitalisation; readmission within 30 days was considered as a distinct hospitalisation. As reported in [19], costs were extracted directly from SNDS and reported in 2020 EUR (see the full methodology in [19]).

We estimated the difference in the median of HCRU-associated costs 1 year before and after hospitalisation with whooping cough among adults by chronic conditions included in the study and by age. For this analysis, we included only patients who were alive at least 1 year after hospitalisation and limited to 2012 and 2019.

## Results

### Epidemiology and cumulative incidence by age and chronic condition

Between 2008 and 2020, 10,893 patients were hospitalised with whooping cough in France: 7,212 were children and adolescents (aged < 18 years) and 3,681 were adults (aged ≥ 18 years) [19]. The most common chronic conditions were asthma and/or COPD (n = 3,560; 32.7%) and immunocompromising conditions (n = 662; 6.1%). Among children and adolescents, 1,263 (17.5%) had asthma and 68 (0.9%) had immunocompromising conditions (Table 1). Among the adults, 2,297 (62.4%) had asthma and/or COPD, 594 (16.1%) had immunocompromising conditions and 454 (12.3%) had diabetes (Table 2).

**Table 1:** Frequency and cumulative incidence of children and adolescents hospitalised with whooping cough, by age and chronic condition, France, 2008–2020 (n = 7,212)

Chronic condition^a^	< 1 year			1–5 years			6–11 years			12–17 years			All		
	Chronic condition (n)	Hospitalised (n)	Cumulative incidence^b^	Chronic condition (n)	Hospitalised (n)	Cumulative incidence^b^	Chronic condition (n)	Hospitalised (n)	Cumulative incidence^b^	Chronic condition (n)	Hospitalised (n)	Cumulative incidence^b^	Chronic condition (n)	Hospitalised (n)	Cumulative incidence^b^
Asthma															
Yes	609,209	750	123.1	6,061,956	265	4.4	4,053,957	160	3.9	2,509,757	88	3.5	13,234,879	1,263	9.5
No	9,548,359	5,447	57.0	45,189,732	294	0.7	59,597,304	117	0.2	60,738,129	91	0.1	175,073,524	5,949	3.4
Immunocompromising conditions^c^															
Yes	14,671	24	163.6	100,198	13	13.0	168,130	14	8.3	219,172	17	7.8	502,171	68	13.5
No	10,142,897	6,173	60.9	51,151,490	546	1.1	63,483,131	263	0.4	63,028,714	162	0.3	187,806,232	7,144	3.8
Diabetes^d^															
Yes	≤ 10			≤ 10			≤ 10			≤ 10			230,272	≤ 10	NA
No	6,187–6,197			549–559			267–277			169–179			188,078,131	7,202–7,212	3.8

EGB: Echantillon Généraliste de Bénéficiaires; NA: not applicable.

^a^ Denominators obtained from EGB.

^b^ Per 100,000 inhabitants.

^c^ Conditions included: asplenia, hereditary immune deficiency, human immunodeficiency virus (HIV)/acquired immunodeficiency syndrome (AIDS), solid tumours, malignant haemopathies, solid organ transplantation, haematopoietic stem cell transplantation, autoimmune or non-respiratory chronic inflammatory diseases treated with immunosuppressants or corticosteroids, and nephrotic syndrome.

^d^ Range presented instead of precise numbers due to data privacy requirements.

**Table 2:** Frequency and cumulative incidence of adults hospitalised with whooping cough, by age and chronic condition, France, 2008–2020 (n = 3,681)

Chronic condition^a^	18–25 years			26–49 years			50–64 years			≥ 65 years			All		
	Chronic condition (n)	Hospitalised (n)	Cumulative incidence^b^	Chronic condition (n)^a^	Hospitalised (n)	Cumulative incidence^b^	Chronic condition (n)^a^	Hospitalised (n)	Cumulative incidence^b^	Chronic condition (n)^a^	Hospitalised (n)	Cumulative incidence^b^	Chronic condition (n)^a^	Hospitalised (n)	Cumulative incidence^b^
Asthma and/or COPD															
Yes	4,633,282	140	3.0	20,188,439	653	3.2	15,352,277	515	3.4	18,408,912	989	5.4	58,582,910	2,297	3.9
No	77,053,239	130	0.2	244,942,477	478	0.2	148,744,294	282	0.2	136,853,902	494	0.4	607,593,914	1,384	0.2
Immunocompromising conditions^c^															
Yes	472,650	20	4.2	5,279,647	133	2.5	10,034,844	164	1.6	20,998,751	277	1.3	36,785,892	594	1.6
No	81,213,871	250	0.3	259,851,269	998	0.4	154,061,727	633	0.4	134,264,063	1,206	0.9	629,390,932	3,087	0.5
Diabetes															
Yes	229,718	≤ 10^d^	NA	3,253,223	44–54^d^	1.4–1.7^d^	11,026,610	103	0.9	20,692,376	297	1.4	35,201,927	454	1.3
No	81,456,803	260–270^d^	0.3	261,877,693	1,077–1,087^d^	0.4	153,069,961	694	0.5	134,570,438	1,186	0.9	630,974,897	3,227	0.5

COPD: chronic obstructive pulmonary disease; EGB: Echantillon Généraliste de Bénéficiaires; ICD-10: International Classification of Diseases, 10th revision; NA: not applicable.

^a^ Denominators obtained from EGB.

^b^ Per 100,000 inhabitants.

^c^ Conditions included: asplenia, hereditary immune deficiency, human immunodeficiency virus (HIV)/acquired immunodeficiency syndrome (AIDS), solid tumours, malignant haemopathies, solid organ transplantation, haematopoietic stem cell transplantation, autoimmune or non-respiratory chronic inflammatory diseases treated with immunosuppressants or corticosteroids and nephrotic syndrome.

^d^ Range presented instead of precise numbers due to data privacy requirements.

In children and adolescents with chronic conditions, the highest cumulative incidence of hospitalisation with pertussis was among those with immunocompromising conditions (13.5/100,000 children and adolescents) followed by those with asthma (9.5/100,000 children and adolescents). Infants with immunocompromising conditions had the highest cumulative incidence overall, 163.6 per 100,000 infants (Table 1).

Among adult patients, the cumulative incidence of hospitalisation with whooping cough was highest among those with asthma and/or COPD: 3.9 per 100,000 inhabitants, varying between 3.0 and 5.4 per 100,000 inhabitants by age group, with the highest cumulative incidence among those aged ≥ 65 years (Table 2). Among patients with diabetes, the cumulative incidence was 1.3 per 100,000 inhabitants. In patients with immunocompromising conditions, the cumulative incidence was 1.6 per 100,000 inhabitants varying from 4.2 in 18–25-year-olds to 1.3 per 100,000 inhabitants in ≥ 65-year-olds.

Stratified cumulative incidences by *Bordetellae* species showed that *B. pertussis* was the most common pathogen, as presented in Supplementary Table S1. The cumulative incidence estimates of hospitalised cases with whooping cough by severity and age (0–17 years, ≥ 18 years, ≥ 65 years) in the various chronic conditions are presented in Supplementary Table S1.

The results for adult patients with only asthma or COPD were aligned with the combined group (asthma and/or COPD) and can be found in Supplementary Table S2. A sensitivity analysis using different denominators for chronic conditions estimating the cumulative incidence in children and adolescents, all adults, those ≥ 65 years and all ages is shown in Supplementary Table S3.

### Cumulative incidence ratios by age and chronic condition

Cumulative incidence ratios were higher among patients with chronic conditions than among those without (Table 3). The incidence of hospitalisation with whooping cough was higher in children and adolescents with chronic conditions than in those without: adjusted CIR was 2.8 (95% confidence interval (CI): 2.6–2.9) in patients with asthma and 3.6 (95% CI: 2.8–4.6) in those with immunocompromising conditions. In adults, the highest adjusted CIR for hospitalisations was in patients with asthma and/or COPD (adjusted CIR = 18.6; 95% CI: 17.4–19.9), followed by immunocompromising conditions (adjusted CIR: 3.6; 95% CI: 3.3–4.0). Adult patients with asthma and/or COPD had a higher adjusted CIR (18.6; 95% CI: 17.4-19.9) than children and adolescents (2.8; 95% CI: 2.6–2.9). When CIRs were calculated for only whooping cough due to *B. pertussis* (A37.1 or A39.0 ICD-10 codes), the point estimates were similar, as presented in Supplementary Table S4.

**Table 3:** Cumulative incidence ratios of hospitalisation with whooping cough, by chronic condition, France, 2008–2020 (n = 10,893)

Chronic condition	Children and adolescents				Adults				
	Cumulative incidence^a^	Crude CIR^a^	Adjusted CIR^b^	95% CI	Cumulative incidence^a^	Crude CIR^a^	Adjusted CIR^b^		95% CI
Asthma and/or COPD^c^									
Yes	9.5	2.8	2.8	2.6–2.9	3.9	19.5	18.6		17.4–19.9
No	3.4	Reference			0.2	Reference			
Immunocompromising conditions									
Yes	13.5	3.6	3.6	2.8-4.6	1.6	3.2	3.6		3.3–4.0
No	3.8	Reference			0.5	Reference			
Diabetes									
Yes	Not analysed				1.3	2.6		2.7	2.5–3.0
No	3.8	Reference			0.5	Reference			

CI: confidence interval; CIR: cumulative incidence ratio; COPD: chronic obstructive pulmonary disease.

^a^ Per 100,000 inhabitants.

^b^ Computed from negative binomial regression models and adjusted on the cyclical pattern of whooping cough.

^c^ In adult patients, asthma and/or COPD were included. In children and adolescents, only asthma was included.

Cumulative incidences and CIRs stratified by age groups are presented in Supplementary Table S5 for children and adolescents (< 1 year, 1–5 years, 6–11 years, 12–17 years) and in Supplementary Table S6 for adults (18–25 years, 26–49 years, 50–64 years and ≥ 65 years). Results for asthma or COPD separately for adults are presented in Supplementary Table S7.

### Costs

Costs associated with hospitalisation of patients with whooping cough between 2011 and 2020 differed by chronic condition and disease severity. Patients with immunocompromising conditions had the highest median overall costs of hospitalisation (EUR 4,401; interquartile range (IQR): 2,577–7,469), followed by those with diabetes (EUR 4,080; IQR: 2,774–5,630). Hospitalisation costs were higher for severe cases than for non-severe cases. For instance, median cost of hospitalisation of severe cases in children and adolescents with asthma was EUR 4,493 (IQR: 2,291–6,856) and median cost of non-severe cases was EUR 2,291 (IQR: 1,010–3,912). Also, median costs of hospitalisation in patients with immunocompromising conditions were higher for children and adolescents than for adults (EUR 5,507 vs EUR 4,273).

In adults overall, HCRU-associated costs, including visits to general practitioners and costs of medications, were similar 1 year before and after being hospitalised with whooping cough (Figure, Table 4). The median difference in the HCRU-associated costs for adult whooping cough patients with asthma and/or COPD was EUR −3, EUR −69 for those with diabetes and EUR +30 for those aged ≥ 65 years. In these calculations, as mentioned in the methodology, we only included patients living 1 year after hospitalisation. For example, only 78.1% (n = 705) of 903 adult patients aged ≥ 65 years were included.

**Figure:**
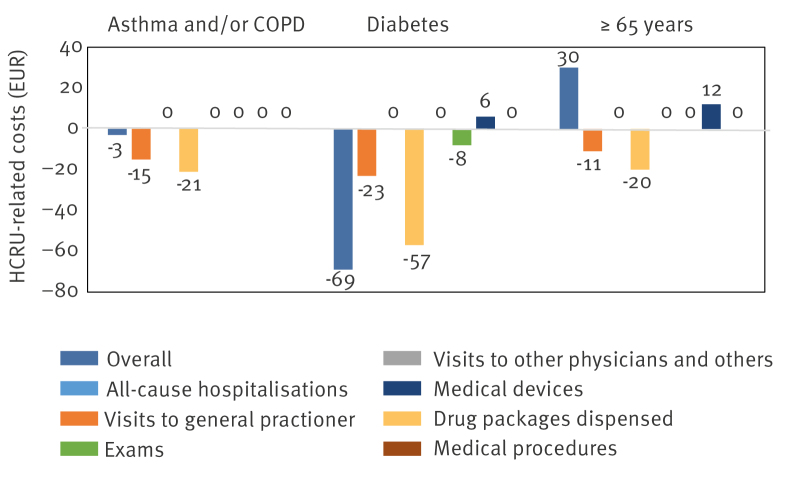
Difference in median healthcare resource utilisation costs for adults 1 year before and after hospitalisation with whooping cough, France, 2012–2019^a^

COPD: chronic obstructive pulmonary disease; HCRU: healthcare resource utilisation.

^a^ Number of patients included: asthma and/or COPD (n = 1,198), diabetes (n = 212) and adults aged ≥ 65 years (n = 705).

**Table 4:** Hospitalisation costs of whooping cough, by chronic condition and disease severity, France, 2011–2020 (n = 3,563)

Characteristic	Children and adolescents			Adults			All		
	Severe cases^a^	Non-severe cases	All	Severe cases^a^	Non-severe cases	All	Severe cases^a^	Non-severe cases	All
Asthma and/or COPD^b^									
Cases (n)	338	646	984	425	1,055	1,480	919	1,921	2,840
Costs (EUR)									
Median	4,493	2,291	2,339	3,542	2,821	2,968	3,965	2,427	2,774
Q1–Q3	2,291–6,856	1,010–3,912	1,599–4,564	934–5,824	1,933–4,255	1,823–4,452	2,126–6,360	1,894–4,186	1,932–4,522
Range	0–63,704	0–13,566	0–63,704	0–157,716	0–21,013	0–157,716	0–157,716	0–21,013	0–157,716
Immunocompromising conditions									
Cases (n)	24	32	56	103	274	377	127	306	433
Costs (EUR)									
Median	9,683	4,300	5,507	7,020	3,960	4,273	7,820	3,984	4,401
Q1–Q3	4,875–38,013	776–6,671	2,284–11,145	3,751–15,158	2,290–5,307	2,677–6,856	4,004–16,660	2,206–5,544	2,577–7,469
Range	697–279,557	327–20,867	327–279,557	0–64,723	0–35,689	0–64,723	0–279,557	0–35,689	0–279,557
Diabetes									
Cases (n)	≤ 10			89–99^c^	181–191^c^	280–290^c^	99	191	290
Costs (EUR)									
Median	NA			4,646	3,707	4,092	4,673	3,692	4,080
Q1–Q3				3,271–8,876	2,668–4,565	2,791–5,630	3,271–9,072	2,668–4,559	2,774–5,630
Range				0–46,671	0–16,648	0–46,671	0–279,557	0–16,648	0–279,557

COPD: chronic obstructive pulmonary disease; EUR: euro; Q: quartile. NA: not applicable.

^a^ Severe cases were patients hospitalised with whooping cough requiring admission to resuscitation, intensive care, continuous monitoring or short-stay emergency observation units, or who died during the hospital stay.

^b^ In adult patients, asthma and/or COPD were included. In children and adolescents, only asthma was included. In the columns for All cases, paediatric patients with asthma and/or COPD are included. Median, Q1–Q3 and range costs are provided in EUR.

^c^ Range presented instead of precise numbers due to data privacy requirements.

Detailed HCRU per chronic condition and costs stratified by severity, age in subjects with asthma or COPD are presented in Supplementary Tables S8 and S9.

## Discussion

This retrospective study on whooping cough in France between 2008 and 2020 complements our previous publication from the same dataset by including chronic conditions [19]. To our knowledge, this is the first comprehensive assessment of hospitalised whooping cough (regardless of *Bordetella* species) patients with chronic conditions, addressing a knowledge gap especially in children and adolescents. Patients with chronic health conditions, regardless of age, particularly respiratory conditions asthma and/or COPD, had higher cumulative incidences for hospitalisations with whooping cough than those without these conditions. Our results (including all *Bordetella* species) are consistent with previous reports focusing mostly on *B. pertussis* [8,12,13,15,21-26] which suggest that chronic conditions could be considered as risk factors for whooping cough in children, adolescents and adults.

The adjusted CIR among adult patients hospitalised with whooping cough with pre-existing asthma and/or COPD was 18.6 times higher than in adults without asthma and/or COPD. It was also 6.6-fold higher than in children and adolescents with asthma. In other studies, chronic respiratory conditions, such as asthma and COPD, have been associated with a higher incidence of whooping cough disease due to *B. pertussis* [12,15,24,27,28]. All cases in our study were hospitalised, which may have resulted in a stronger association with whooping cough and chronic respiratory conditions. A previous study reported augmented incidence of COPD and asthma exacerbations around whooping cough diagnosis [26].

Aligned with current evidence, our results support the need for vaccination against *B. pertussis* in individuals with chronic respiratory conditions as most cases of whooping cough are caused by vaccine-preventable *B. pertussis* [2,29-31]. This is particularly important considering that age-related chronic conditions, such as COPD, are expected to increase in high-income countries with the rising life expectancy [32]. According to a modelling study conducted in France, the prevalence of COPD in adults aged ≥ 45 years by 2025 was estimated at 9.6%, translating to 2,801,605 patients [33]. In the present study, 17.5% of children and adolescents hospitalised with whooping cough had asthma and more than 60% of adults had asthma and/or COPD. For such patients it is essential to ensure that vaccinations are up to date and administered according to recommended guidelines.

Overall, the chronic condition with the highest median cost of hospitalised pertussis in France between 2011 and 2020 was immunocompromising conditions (EUR 4,401), 7.9% costlier than diabetes (EUR 4,080) and 58.7% costlier than asthma and/or COPD (EUR 2,774). The highest costs were for children and adolescents with an immunocompromising condition (EUR 5,507). In 2011–2020, the median cost for hospitalisation of a patient with whooping cough was EUR 3,259 [19], which is lower than the median cost for patients with immunocompromising conditions and diabetes but higher than for asthma and/or COPD of our study. This is partially explained as the age group with the highest cost were infants aged < 2 months (median cost: EUR 4,880), a population with no risk of COPD and lower risk of asthma. As expected, costs for severe cases were higher than for non-severe, as also seen in the general population [19]. However, these differences between costs for severe and non-severe whooping cough were more pronounced in children and adolescents than in adults. This was also seen in the previous study, with the difference in costs of severe and non-severe disease being larger in infants than in other age groups [19]. The differences (range: EUR −69 to 30) in HCRU-related costs for pre- and post-hospitalisation with whooping cough were small in adults with asthma and/or COPD, diabetes and aged ≥ 65 years. However, these results need to be interpreted with caution as only hospitalised cases were included, and no costs could be calculated for those who did not survive.

This study has some limitations. We used SNDS data which do not include the results of laboratory tests; hence, whether the cases were laboratory-confirmed. However, as these cases were hospitalised, either a clinical suspicion or laboratory confirmation was likely. We did not address the differences in testing or suspicion rates among individuals with and without chronic conditions. Some whooping cough cases were not likely included in the study due to underdiagnosis which may underestimate the incidence. In addition, as only hospitalised cases were included, the incidence of whooping cough is probably higher. We used non-validated algorithms for capturing the chronic conditions included. Some conditions of interest like obesity or smoking status could not be properly captured in SNDS leading to underestimation and selection bias towards more severe cases. These results were not presented and would warrant specific studies for better case ascertainment. The methodology used for calculating the denominators of cumulative incidences might have under- or overestimated the population at risk (census data providing data for 5-year age bands, the use of EGB, extrapolation coefficient). Given the absence of available prevalence data for the chronic conditions and age groups of interest, external calibrators or alternative denominators could not be included. Also, we only assessed the incidences and costs of whooping cough hospitalisations in separate subgroups with separate chronic conditions and did not investigate the impact of multimorbidity. While the CIRs reported in our study were adjusted for cyclic pattern, other factors, such as age as continuous/stratified, sex, region, vaccination status, year fixed effects, secular trends, testing intensity were not accounted for in this analysis. Finally, we used a broad definition for severe cases, including those that were followed up in emergency care, which provides a sensitive but less specific definition which could overestimate the incidence in severe cases and underestimate severe costs.

## Conclusion

Incidences of whooping cough-related hospitalisation were higher among patients with asthma and/or COPD in adults and immunocompromising conditions in children and adolescents. Thus, these findings emphasise the need for whooping cough awareness among patients with chronic conditions and their healthcare professionals to improve diagnosis, and prevention through vaccination. Prevention of whooping cough in adults could be strengthened by using booster vaccines including protection against *B. pertussis*, in accordance with the respective national immunisation schedules, particularly among older adults with underlying conditions who are at increased risk of severe and fatal disease.

## Data Availability

The data supporting the study findings are part of the National health data system (SNDS, Système national des Données de Santé) and are available from the CNAM (Caisse Nationale de L’assurance Maladie (French National Health Insurance Fund)) server on a study data folder. Restrictions apply to the availability of these data and the code used for their analysis, which were used with a special permission for this study. Further details on Sanofi’s data sharing criteria, eligible studies, and process for requesting access can be found at https://www.vivli.org/.

## Supplementary Data

631000 Supplementary Material
