# Supplementary material for: Whooping cough hospitalisations among patients with chronic conditions: a retrospective analysis of incidence and costs, France, 2008 to 2020
Source: Euro Surveill. 2026 Aug 13;31(32):2500561. doi: 10.2807/1560-7917.ES.2026.31.32.2500561 (PMC13476727; doi:10.2807/1560-7917.ES.2026.31.32.2500561)
Supplement: Supplementary file 1 [file 25-00561_VARGAS-ZAMBRANO_Supplement.pdf]

Title: Whooping cough hospitalisations among patients with chronic conditions: a retrospective analysis of incidence and costs, France, 2008 to 2020

This supplementary material is hosted by *Eurosurveillance* as supporting information alongside the article **Whooping cough hospitalisations among patients with chronic conditions: a retrospective analysis of incidence and costs, France, 2008 to 2020**, on behalf of the authors, who remain responsible for the accuracy and appropriateness of the content. The same standards for ethics, copyright, attributions and permissions as for the article apply. Supplements are not edited by *Eurosurveillance* and the journal is not responsible for the maintenance of any links or email addresses provided therein

Supplementary Table S1: Cumulative incidence estimates of hospitalised whooping cough cases between 2008 and 2020 in France for children, adolescents, adults, ≥65 years and all ages according to severity and *Bordetella* species

			**All cases**		**Severe cases**		**Non-severe cases**		***B. pertussis***		**Other *Bordetella* species**	
	**Chronic condition**	***N***	***n***	**CI[a]**	***n***	**CI[a]**	***n***	**CI[a]**	***n***	**CI[a]**	***n***	**CI[a]**
**0–17 years**	**Asthma**											
	Yes	13,234,879	1,263	9.5	414	3.1	849	6.4	1,177	8.9	86	0.6
	No	175,073,524	5,949	3.4	2,424	1.4	3,525	2.0	5,582	3.2	367	0.2
	**Diabetes**											
	Yes	230,272	≤10	-	≤10	-	≤10	-	≤10	-	≤10	-
	No	188,078,131	7,202-7,212[b]	3.8	2,828-2,838[b]	1.5	4,364-4,374[b]	2.3	6,749-6,759[b]	3.6	443-453[b]	0.2
	**Immunocompromising condition**											
	Yes	502,171	68	13.5	25	5.0	43	8.6	57-68[b]	11.4-13.5[b]	≤10	-
	No	187,806,232	7,144	3.8	2,813	1.5	4,331	2.3	6,691-6,701[b]	3.6	443-453[b]	0.2
**≥18 years**	**Asthma**											
	Yes	31,026,313	1,559	5.0	391	1.3	1,168	3.8	1,442	4.6	117	0.4
	No	635,150,511	2,122	0.3	517	0.1	1,605	0.3	1,957	0.3	165	0.0

	**COPD**											
	Yes	7,735,765	558	7.2	137	1.8	421	5.4	501	6.5	57	0.7
	No	658,441,059	3,123	0.5	771	0.1	2,352	0.4	2,898	0.4	225	0.0
	**Asthma and/or COPD**											
	Yes	58,582,910	2,297	3.9	570	1.0	1,727	2.9	2,112	3.6	185	0.3
	No	607,593,914	1,384	0.2	338	0.1	1,046	0.2	1,287	0.2	97	0.0
	**Diabetes**											
	Yes	35,201,927	451-461[b]	1.3	117-127[b]	0.3-0.4[b]	324-334[b]	0.9	414-424[b]	1.2	27-37[b]	0.1
	No	630,974,897	3,220-3,230[b]	0.5	781-791[b]	0.1	2,439-2,449[b]	0.4	2,975-2,985[b]	0.5	245-255[b]	0.0
	**Immunocompromising condition**											
	Yes	36,785,892	594	1.6	126	0.3	468	1.3	527-537[b]	1.4-1.5[b]	57-67[b]	0.2
	No	629,390,932	3,087	0.5	782	0.1	2,305	0.4	2,863-2,873[b]	0.5	215-225[b]	0.0
**≥65 years**	**Asthma**											
	Yes	9,916,517	639	6.4	143	1.4	496	5.0	582	5.9	57	0.6
	No	145,346,297	844	0.6	210	0.1	634	0.4	769	0.5	75	0.1
	**COPD**											
	Yes	4,552,588	367	8.1	78	1.7	289	6.3	329	7.2	38	0.8
	No	150,710,226	1,116	0.7	275	0.2	841	0.6	1,022	0.7	94	0.1
	**Asthma and/or COPD**											
	Yes	18,408,912	989	5.4	221	1.2	768	4.2	899	4.9	90	0.5
	No	136,853,902	494	0.4	132	0.1	362	0.3	452	0.3	42	0.0
	**Diabetes**											
	Yes	20,692,376	297	1.4	78	0.4	219	1.1	275	1.3	22	0.1
	No	134,570,438	1,186	0.9	275	0.2	911	0.7	1,076	0.8	110	0.1
	**Immunocompromising condition**											
	Yes	20,998,751	277	1.3	63	0.3	214	1.0	240	1.1	37	0.2

	No	134,264,063	1,206	0.9	290	0.2	916	0.7	1,111	0.8	95	0.1
**All**	**Asthma**										
	Yes	44,261,192	2,822	6.4	805	1.8	2,017	4.6	2,619	5.9	203	0.5
	No	810,224,035	8,071	1.0	2,941	0.4	5,130	0.6	7,539	0.9	532	0.1
	**COPD[c]**										
	Yes	7,769,890	570	7.3	144	1.9	426	5.5	510	6.6	60	0.8
	No	846,715,337	10,323	1.2	3,602	0.4	6,721	0.8	9,648	1.1	675	0.1
	**Asthma and/or COPD[c]**										
	Yes	78,444,264	4,034	5.1	1,160	1.5	2,874	3.7	3,731	4.8	303	0.4
	No	776,040,963	6,859	0.9	2,586	0.3	4,273	0.6	6,427	0.8	432	0.1
	**Diabetes**										
	Yes	35,432,199	451-461[b]	1.3	127	0.4	334	0.9	424	1.2	37	0.1
	No	819,053,028	10,422-10,432[b]	1.3	3,619	0.4	6,813	0.8	9,734	1.2	698	0.1
	**Immunocompromising condition**										
	Yes	37,288,063	662	1.8	151	0.4	511	1.4	595	1.6	67	0.2
	No	817,197,164	10,231	1.3	3,595	0.4	6,636	0.8	9,563	1.2	668	0.1

N, number of subjects with chronic condition; n, number of patients hospitalized with whooping cough; CI, cumulative incidence; COPD, chronic obstructive pulmonary disease; EGB, Echantillon Généraliste de Bénéficiaires; ICD-10, International Classification of Diseases, Tenth Revision.

[a]Per 100,000 inhabitants, [b]Data masked to prevent secondary derivation of cells with values ≤ 10, [c]Include cases in children and adolescents not reported separately for this age-group

Bordetella species: Patients with at least one diagnosis referring to an ICD-10 code starting with A37.0 (Bordetella pertussis) or A37.9 (unspecified Bordetella species).

Other Bordetella species: Patients with at least one diagnosis referring to an ICD-10 code starting with A37.1 or A37.8 (and without A37.0 or A37.9)

Supplementary Table S2: **Cumulative incidence estimates of hospitalized with whooping cough cases between 2008 and 2020 in France, for adults with asthma or COPD in adults stratified by age-group**

Chronic condition[a]	18–25 years			26–49 years			50–64 years			≥65 years		
	All cases			All cases			All cases			All cases		
	N	***n***	**CI[b]**	***N***	***n***	**CI[b]**	***N***	***n***	**CI[b]**	***N***	***n***	**CI[b]**
**Asthma**												
Yes	2,344,890	94	4.0	10,635,072	469	4.4	8,129,834	357	4.4	9,916,517	639	6.4
No	79,341,631	176	0.2	254,495,844	662	0.3	155,966,737	440	0.3	145,346,297	844	0.6
**COPD**												
Yes	43,942	≤10	-	780,669	46-56[c]	5.9-7.2[c]	2,358,566	131	5.6	4,552,588	367	8.1
No	81,642,579	260-270[c]	0.3	264,350,247	1,075-1,085[c]	0.4	161,738,005	666	0.4	150,710,226	1,116	0.7

N, number of subjects with chronic condition; n, number of patients hospitalized with whooping cough;CI, cumulative incidence; COPD, chronic obstructive pulmonary disease; ICD-10, International Classification of Diseases, Tenth Revision; *N*, number of inhabitants with chronic condition; *n*, number of whooping cough hospitalisation

[a]Denominators obtained from EGB; [b]Per 100,000 inhabitants, [c]Data masked to prevent secondary derivation of cells with values ≤ 10

Supplementary Table S3: Cumulative incidence estimates of hospitalised whooping cough cases between 2008 and 2020 in France for children, adolescents, adults, ≥65 years and all ages using sensitivity analyses on the prevalence of the chronic conditions

		All cases			Severe cases		Non-severe cases		*B. pertussis*		Other *Bordetella* species	
	Chronic condition [a]	***N***	***n***	CI[b]	***n***	CI[b]	***n***	CI[b]	***n***	CI[b]	***n***	CI[b]
	**Asthma**											
**0–17 years**	Yes	15,220,111	1,263	8.3	414	2.7	849	5.6	1,177	7.7	86	0.6
	No	173,088,292	5,949	3.4	2,424	1.4	3,525	2.0	5,582	3.2	367	0.2
	**Diabetes**											
	Yes	264,813	≤10	-	≤10	-	≤10	-	≤10	-	≤10	-
	No	188,043,590	7,202-7,212[c]	3.8	2,828-2,838[c]	1.5	4,364-4,374[c]	2.3	6,749-6,759[c]	3.6	443-453[c]	0.2
	**Immunocompromising condition**											
	Yes	652,822	68	10.4	25	3.8	43	6.6	58-68[c]	8.9-10.4[c]	≤10	-
	No	187,655,581	7,144	3.8	2,813	1.5	4,331	2.3	6,693	3.6	451	0.2
**≥18 years**	**Asthma**											
	Yes	35,680,260	1,559	4.4	391	1.1	1,168	3.3	1,442	4.0	117	0.3
	No	630,496,564	2,122	0.3	517	0.1	1,605	0.3	1,957	0.3	165	0.0
	**COPD**											
	Yes	10,056,495	558	5.5	137	1.4	421	4.2	501	5.0	57	0.6
	No	656,120,330	3,123	0.5	771	0.1	2,352	0.4	2,898	0.4	225	0.0
	**Asthma and/or COPD**											
	Yes	76,157,783	2,297	3.0	570	0.7	1,727	2.3	2,112	2.8	185	0.2
	No	590,019,041	1,384	0.2	338	0.1	1,046	0.2	1,287	0.2	97	0.0
	**Diabetes**											
	Yes	40,482,216	451-461[c]	1.1	117-127[c]	0.3	324-334[c]	0.8	414-424[c]	1.0	27-37[c]	0.1
	No	625,694,608	3,220-3,230[c]	0.5	781-791[c]	0.1	2,439-2,449[c]	0.4	2,975-2,985[c]	0.5	245-255[c]	0.0
	**Immunocompromising condition**											
	Yes	47,821,660	594	1.2	126	0.3	468	1.0	529	1.1	65	0.1
	No	618,355,164	3,087	0.5	782	0.1	2,305	0.4	2,870	0.5	217	0.0

**≥65 years**	**Asthma**											
	Yes	11,403,995	639	5.6	143	1.3	496	4.3	582	5.1	57	0.5
	No	143,858,819	844	0.6	210	0.1	634	0.4	769	0.5	75	0.1
	**COPD**											
	Yes	5,918,364	367	6.2	78	1.3	289	4.9	329	5.6	38	0.6
	No	149,344,450	1,116	0.7	275	0.2	841	0.6	1,022	0.7	94	0.1
	**Asthma and/or COPD**											
	Yes	23,931,586	989	4.1	221	0.9	768	3.2	899	3.8	90	0.4
	No	131,331,228	494	0.4	132	0.1	362	0.3	452	0.3	42	0.0
	**Diabetes**											
	Yes	23,796,232	297	1.2	78	0.3	219	0.9	275	1.2	22	0.1
	No	131,466,582	1,186	0.9	275	0.2	911	0.7	1,076	0.8	110	0.1
	**Immunocompromising condition**											
	Yes	27,298,376	277	1.0	63	0.2	214	0.8	240	0.9	37	0.1
	No	127,964,438	1,206	0.9	290	0.2	916	0.7	1,111	0.9	95	0.1
**All**	**Asthma**											
	Yes	50,900,371	2,822	5.5	805	1.6	2,017	4.0	2,619	5.1	203	0.4
	No	803,584,856	8,071	1.0	2,941	0.4	5,130	0.6	7,539	0.9	532	0.1
	**COPD[d]**											
	Yes	10,100,857	570	5.6	134-144[c]	1.4	416-426[c]	4.2	500-510[c]	5.0	50-60[c]	0.6
	No	844,384,370	10,323	1.2	3,602	0.4	6,721	0.8	9,648	1.1	675	0.1
	**Asthma and/or COPD**											
	Yes	101,977,543	4,034	4.0	1,160	1.1	2,874	2.8	3,731	3.7	303	0.3
	No	752,507,684	6,859	0.9	2,586	0.3	4,273	0.6	6,427	0.9	432	0.1
	**Diabetes**											
	Yes	40,747,029	451-461[c]	1.1	127	0.3	334	0.8	424	1.0	37	0.1
	No	813,738,198	10,422-10,432[c]	1.3	3,619	0.4	6,813	0.8	9,734	1.2	698	0.1
	**Immunocompromising condition**											
	Yes	48,474,482	662	1.4	151	0.3	511	1.1	595	1.2	67	0.1
	No	806,010,745	10,231	1.3	3,595	0.4	6,636	0.8	9,563	1.2	668	0.1

N, number of subjects with chronic condition; n, number of patients hospitalized with whooping cough; CI, cumulative incidence; COPD, chronic obstructive pulmonary disease; EGB, Echantillon Généraliste de Bénéficiaires; ICD-10, International Classification of Diseases, Tenth Revision

[a]Denominators obtained from EGB with sensitive analyses (asthma, diabetes: +15%; COPD, asthma/COPD, immunocompromising conditions: +30%); [b]Per 100,000 inhabitants. [c]Data masked to prevent secondary derivation of cells with values ≤ 10, [d]Include cases in children and adolescents not reported separately for this age-group

Bordetella species: Patients with at least one diagnosis referring to an ICD-10 code starting with A37.0 (*Bordetella pertussis*) or A37.9 (unspecified *Bordetella* species).
Other *Bordetella* species: Patients with at least one diagnosis referring to an ICD-10 code starting with A37.1 or A37.8 (and without A37.0 or A37.9).

Supplementary Table S4: Comparison of cumulative incidence ratios of hospitalised whooping cough due to *B. pertussis* between 2008 and 2020 in France for children and adolescents and adults with chronic conditions

**Chronic condition**	**Children and adolescents**				**Adults**			
	**CI[a]**	**Crude CIR[a]**	**Adjusted CIR[b]**	**95% CI**	**CI[a]**	**Crude CIR[a]**	**Adjusted CIR[b]**	**95% CI**
Asthma and/or COPD[c]								
Yes	8.9	2.8	2.7	2.6-2.9	3.6	18.0	18.5	17.2-19.8
No	3.2	Reference			0.2	Reference		
Diabetes								
Yes	Not analysed				1.2	2.4	2.7	2.5-3.0
No	3.6	Reference			0.5	Reference		
Immunocompromising conditions								
Yes	13.1	3.6	4.0	3.1-5.0	1.4	2.8	3.5	3.2-3.8
No	3.6	Reference			0.5	Reference		

Patients with at least one diagnosis referring to an ICD-10 code starting with A37.0 (*Bordetella pertussis*) or A37.9 (unspecified *Bordetella* species)

CI: cumulative incidence; CIR: cumulative incidence ratio; COPD: chronic obstructive pulmonary disease.
[a] Per 100,000 inhabitants.
[b] Computed from negative binomial regression models and adjusted on the cyclical pattern of whooping cough.
[c] In adult patients, asthma and/or COPD were included. In children and adolescents, only asthma was included.

Supplementary Table S5: Comparison of cumulative incidence ratios of hospitalised whooping cough cases between 2008 and 2020 in France for children and adolescents with chronic conditions

	<1 year			1–5 years			6–11 years			12–17 years		
	All cases			All cases			All cases			All cases		
**Chronic condition**	**CI[a]**	**Crude CIR**	**Adjusted CIR[b] (95%CI)**	**CI[a]**	**Crude CIR**	**Adjusted CIR[b] (95%CI)**	**CI[a]**	**Crude CIR**	**Adjusted CIR[b] (95%CI)**	**CI[a]**	**Crude CIR**	**Adjusted CIR[b] (95%CI)**
**Asthma**												
Yes	123.1	2.2	2.1 (2.0, 2.3)	4.4	6.3	6.6 (5.6, 7.8)	3.9	19.5	19.9 (15.7, 25.3)	3.5	35.0	23.5 (17.5, 31.5)
No	57.0	Ref	Ref	0.7	Ref	Ref	0.2	Ref	Ref	0.1	Ref	Ref
**Immunocompromising condition**												
Yes	163.6	2.7	3.7 (2.5, 5.5)	13.0	11.8	19.2 (11.0, 33.4)	8.3	20.8	25.8 (15.0, 44.4)	7.8	26.0	44.3 (26.6, 73.6)
No	60.9	Ref	Ref	1.1	Ref	Ref	0.4	Ref	Ref	0.3	Ref	Ref

CI, cumulative incidence; CIR, cumulative incidence ratio.

[a]Per 100,000 inhabitants; [b]Computed from negative binomial regression models and adjusted on the cyclical pattern of the whooping cough disease;

Supplementary Table S6: Comparison of cumulative incidence ratios of hospitalised whooping cough cases between 2008 and 2020 in France for adults with Chronic conditions

	18–25 years			26–49 years			50–64 years			≥65 years		
	All cases			All cases			All cases			All cases		
**Chronic Condition**	**CI[a]**	**Crude CIR**	**Adjusted CIR[b] (95%CI)**	**CI[a]**	**Crude CIR**	**Adjusted CIR[b] (95%CI)**	**CI[a]**	**Crude CIR**	**Adjusted CIR[b] (95%CI)**	**CI[a]**	**Crude CIR**	**Adjusted CIR[b] (95%CI)**
**Asthma and/or COPD**												
Yes	3.0	15.0	19.0 (14.9,24.1)	3.2	16.0	17.3 (15.4,19.5)	3.4	17.0	19.2 (16.6,22.2)	5.4	13.5	16.6 (14.8,18.5)
No	0.2	Ref	Ref	0.2	Ref	Ref	0.2	Ref	Ref	0.4	Ref	Ref
**Immunocompromising condition**												
Yes	4.2	14.0	19.6 (12.4,31.0)	2.5	6.3	7.0 (5.8,8.4)	1.6	4.0	4.2 (3.5,5.0)	1.3	1.4	1.6 (1.4,1.9)
No	0.3	Ref	Ref	0.4	Ref	Ref	0.4	Ref	Ref	0.9	Ref	Ref
**Diabetes**												
Yes	-	-	-	1.5	3.8	3.9 (2.9,5.1)	0.9	1.8	2.1 (1.7,2.6)	1.4	1.6	1.8 (1.6,2.0)
No	0.3	Ref	Ref	0.4	Ref	Ref	0.5	Ref	Ref	0.9	Ref	Ref

CI, cumulative incidence; CIR, cumulative incidence ratio; COPD, chronic obstructive pulmonary disease.

[a]Per 100,000 inhabitants; [b]Computed from negative binomial regression models and adjusted on the cyclical pattern of the whooping cough disease;

Supplementary Table S7: Comparison of cumulative incidence ratios of hospitalised whooping cough cases between 2008 and 2020 in France for adults with either asthma or COPD

	Adults		
	CI[a]	Crude CIR	Adjusted CIR[b] (95%CI)
**Asthma**			
Yes	5.0	16.7	15.8 (14.7,16.9)
No	0.3	Ref	Ref
**COPD**			
Yes	7.2	14.4	16.5 (15.1,18.0)
No	0.5	Ref	Ref

CI, cumulative incidence; CIR, cumulative incidence ratio; COPD, chronic obstructive pulmonary disease.

[a]Per 100,000 inhabitants; [b]Computed from negative binomial regression models and adjusted on the cyclical pattern of the whooping cough disease.

Supplementary Table S8: Comparison of HCRU and HCRU-related costs in the year post-hospitalisation versus pre-hospitalisation in hospitalised whooping cough cases between 2012 and 2019[a] with Chronic condition in France for all adults (≥18 years)

		Diabetes (*N*=212)			Asthma and/or COPD (*N*=1198)			Asthma (*N*=820)			COPD (*N*=269)		
		In the year pre-hospitalisation for whooping cough	In the year post-hospitalisation for whooping cough	Intra-individual difference post- vs. pre-hospitalisation for whooping cough [95% confidence interval]	In the year pre-hospitalisation for whooping cough	In the year post-hospitalisation for whooping cough	Intra-individual difference post vs. pre-hospitalisation for whooping cough [95% confidence interval]	In the year pre-hospitalisation for whooping cough	In the year post-hospitalisation for whooping cough	Intra-individual difference post vs. pre-hospitalisation for whooping cough [95% confidence interval]	In the year pre-hospitalisation for whooping cough	In the year post-hospitalisation for whooping cough	Intra-individual difference post vs. pre-hospitalisation for whooping cough [95% confidence interval]
**Number of HCRU events per patient (*n*)**													
Overall	Median	184	172	-10 [-18, -1]	108	104	-4 [-6, -1]	107	105	-5 [-8, -1]	185	167	-1 [-8, 9]
	Q1–Q3	[127–273]	[104–273]	-	[59–197]	[49–196]	-	[60–197]	[50–195]	-	[107–269]	[100–263]	-
	Min–Max	(0–768)	(0–1050)	-	(0–900)	(0–1114)	-	(0–900)	(0–912)	-	(1–707)	(0–1050)	-
Visits to general practitioner	Median	10	7	-2 [-2,-1]	8	6	-1 [-1,-1]	8	6	-1 [-2, -1]	9	8	-1 [-1,0]
	Q1–Q3	[6–15]	[3–13]	-	[4–12]	[3–10]	-	[4–12]	[3–11]	-	[5–13]	[4–12]	-
	Min–Max	(0–51)	(0–44)	-	(0–59)	(0–90)	-	(0–59)	(0–90)	-	(0–51)	(0–51)	-
Visits to other physicians and	Median	1	1	0 [0,0]	1	1	0 [0,0]	1	1	0 [0,0]	2	2	0 [0,0]
	Q1–Q3	[0–4]	[0–3]	-	[0–4]	[0 –3]	-	[0–4]	[0–3]	-	[0–5]	[0–4]	-

other HCPs	Min–Max	(0–60)	(0–45)		(0–39)	(0–45)		(0–39)	(0–45)	-	(0–31)	(0–45)	-
Drug packages dispensed	Median	134	134	-7 [-12,0]	81	78	-4 [-5, -1]	80	78	-4 [-6, -1]	141	127	0 [-7,6]
	Q1–Q3	[87–206]	[68–214]	-	[41–148]	[33–149]	-	[42–148]	[33–149]	-	[76–213]	[69–202]	-
	Min–Max	(0–532)	(0–777)	-	(0–618)	(0–816)	-	(0–618)	(0–722)	-	(0–618)	(0–777)	-
All-cause hospitalisations	Median	1	1	0 [0,0]	0	1	0 [0,0]	0	1	0 [0,0]	1	1	0 [0,0]
	Q1–Q3	[0–3]	[0–3]	-	[0–1]	[0–2]	-	[0–1]	[0–2]	-	[0–3]	[0–3]	-
	Min–Max	(0 –141)	(0–150)	-	(0–73)	(0–150)	-	(0–73)	(0–150)	-	(0–43)	(0–150)	-
Examinations	Median	10	9	0 [-1,0]	5	5	0 [0,0]	5	5	0 [0,0]	7	6	0 [0,1]
	Q1–Q3	[5–21]	[4–20]	-	[2–12]	[2–13]	-	[2–11]	[2–12]	-	[3–19]	[3–20]	-
	Min–Max	(0–577)	(0–420)	-	(0–401)	(0–830)	-	(0–401)	(0–221)	-	(0–233)	(0–420)	-
Medical devices	Median	10	11	0 [-1,0]	3	3	0 [0,0]	3	4	0 [0,0]	6	6	0 [0,1]
	Q1–Q3	[3–19]	[3–20]	-	[1–7]	[1–9]	-	[1–8]	[1–9]	-	[2–14]	[2–18]	-
	Min–Max	(0–134)	(0–125)	-	(0–146)	(0–161)	-	(0–146)	(0–125)	-	(0–134)	(0–133)	-
Medical procedure	Median	0	0	0 [0,0]	0	0	0 [0,0]	0	0	0 [0,0]	0	0	0 [0,0]
	Q1–Q3	[0–0]	[0–0]	-	[0–0]	[0–0]	-	[0–0]	[0–0]	-	[0–0]	[0–0]	-
	Min–Max	(0–57)	(0–78)	-	(0–59)	(0–119)	-	(0–40)	(0–84)	-	(0–42)	(0–79)	-
**HCRU-related costs per patient (€)**													
Overall	Median	2754	2873	-69 [-180,379]	1054	1078	-3 [-4,118]	983	1013	-13 [-17,131]	2094	2232	43 [-62,1080]

	Q1–Q3	[1323–4867]	[1174–5738]	-	[422–2387]	[409–2649]	-	[435–2278]	[424–2470]	-	[1177–4207]	[1085–5838]	-
	Min–Max	(0–54,768)	(0–73,537)	-	(0–54768)	(0–73,537)	-	(0–50,501)	(0–73,537)	-	(0–50,501)	(0–35,623)	-
Visits to general practitioner	Median	195	150	-23 [-35, -13]	134	108	-15 [-17, -11]	136	114	-15 [-19, -10]	169	151	-6 [-21,0]
	Q1–Q3	[100–315]	[72–288]	-	[70–224]	[45–208]	-	[72–220]	[50–209]	-	[88–285]	[67–254]	-
	Min–Max	(0–1427)	(0–966)	-	(0–1538)	(0–2210)	-	(0–1538)	(0–2210)	-	(0–1150)	(0–2210)	-
Visits to other physicians and other HCPs	Median	36	26	0 [0,0]	32	31	0 [0,0]	32	30	0 [0,0]	45	32	0 [-4,0]
	Q1–Q3	[0–93]	[0–80]	-	[0–88]	[0–78]	-	[0–86]	[0–78]	-	[0–119]	[0–88]	-
	Min–Max	(0–1701)	(0–1158)	-	(-3–1143)	(-4–1288)	-	(-1–1143)	(-4–1288)	-	(-3–1010)	(0–941)	-
Drug packages dispensed	Median	1278	1316	-57 [-119,0]	471	477	-21 [-31, -6]	443	463	-25 [-38, -6]	1087	1071	-34 [-90,21]
	Q1–Q3	[651–2240]	[437–2493]	-	[169–1220]	[147–1243]	-	[180–1151]	[150–1174]	-	[555–2063]	[512–1945]	-
	Min–Max	(0–50,416)	(0–72,758)	-	(0–50,416)	(0–72,758)	-	(0–47,155)	(0–72,758)	-	(0–47,155)	(0–27,860)	-
All-cause hospitalisations	Median	0	0	0 [0,199]	0	0	0 [0,0]	0	0	0 [0,0]	0	0	0 [0,599]
	Q1–Q3	[0–0]	[0–0]	-	[0–0]	[0–0]	-	[0–0]	[0–0]	-	[0–0]	[0–0]	-
	Min–Max	(0–0)	(0–0)	-	(0–0)	(0–0)	-	(0–0)	(0–0)	-	(0–0)	(0–0)	-
Examinations	Median	173	165	-8 [-27,2]	85	78	0 [-4,0]	83	75	0 [-7,0]	134	118	-5 [-21,4]
	Q1–Q3	[84–355]	[59–312]	-	[31–209]	[23–204]	-	[31–200]	[23–199]	-	[53–297]	[39–268]	-
	Min–Max	(0–2351)	(0–2371)	-	(0–2491)	(0–3472)	-	(0–1882)	(0–2888)	-	(0–1586)	(0–2465)	-
Medical devices	Median	376	460	6 [0,29]	28	39	0 [0,6]	30	40	0 [0,4]	117	165	19 [0,41]

	Q1–Q3	[41–964]	[47–1237]	-	[3–176]	[6–294]	-	[6–175]	[6–289]	-	[16–610]	[25–1035]	-
	Min–Max	(0–11,853)	(0–14,965)	-	(0–14,652)	(0–20,731)	-	(0–14,652)	(0–20,731)	-	(0–14,652)	(0–14,965)	-
Medical procedure	Median	0	0	0 [0,0]	0	0	0 [0,0]	0	0	0 [0,0]	0	0	0 [0,0]
	Q1–Q3	[0–0]	[0– 0]	-	[0–0]	[0–0]	-	[0–0]	[0–0]	-	[0–0]	[0–0]	-
	Min–Max	(0–5170)	(0–9884)	-	(0–6165)	(0–9884)	-	(0–6165)	(0–5474)	-	(0–6165)	(0–9884)	-

COPD, chronic obstructive pulmonary disease; HCP, healthcare professional; HCRU, healthcare resource utilisation; *n*, number of HCRU events per person; *N*, number of patients in each group.

[a]HCRU data were collected for a limited period, 2012–2019.

Supplementary Table S9: Costs of whooping cough in patients with either asthma or COPD 2011-2020

	All			Children and adolescents			Adults		
	**Severe cases**	**Non-severe cases**	**All cases**	**Severe cases**	**Non-severe cases**	**All cases**	**Severe cases**	**Non-severe cases**	**All cases**
**Asthma**									
N	621 (100.0)	1379 (100.0)	2000 (100.0)	338 (100.0)	646 (100.0)	984 (100.0)	283 (100.0)	733 (100.0)	1016 (100.0)
Median	3754	2332	2672	4493	2291	2339	3274	2766	2839
Q1–Q3	(2098-6189)	(1723–3974)	(1780–4493)	(2291–6856)	(1010–3912)	(1599–4564)	(759–5389)	(1954–4120)	(1805–4342)
Range	(0; 157,716)	(0; 15,863)	(0; 157,716)	(0; 63,704)	(0; 13,566)	(0; 63,704)	(0; 157,716)	(0; 15,863)	(0; 157,716)
**COPD[a]**									
N	99-109[b] (100.0)	239-249 [b] (100.0)	358 (100.0)	-	-	-	93-103[b] (100.0)	234-244[b] (100.0)	347 (100.0)
Median	4488	3292	3613	-	-	-	4455	3322	3568
Q1–Q3	(2834–8125)	(1703–4630)	(2257–5269)	-	-	-	(2820–7473)	(1699–4624)	(2257–5061)
Range	(0; 64,723)	(0; 15,454)	(0; 64,723)	-	-	-	(0; 64,723)	(0; 15,454)	(0; 64,723)

COPD, chronic obstructive pulmonary disease

[a] Include cases in children and adolescents not reported separately for this age-group

[b]Data masked to prevent secondary derivation of cells with values ≤ 10,
